# Epidemiology of Pediatric Ocular Trauma in the Chaoshan Region, China, 2001–2010

**DOI:** 10.1371/journal.pone.0060844

**Published:** 2013-04-08

**Authors:** He Cao, Liping Li, Mingzhi Zhang, Hongni Li

**Affiliations:** 1 Injury Prevention Research Center, Shantou University Medical College, Shantou, Guangdong, People's Republic of China; 2 Joint Shantou International Eye Centre, Shantou, Guangdong Province, People’s Republic of China; 3 Department of Ophthalmology, Shantou Central Hospital, Shantou, Guangdong Province, People’s Republic of China; University of Utah (Salt Lake City), United States of America

## Abstract

**Background:**

Ocular trauma is the leading cause of monocular visual disability and noncongenital unilateral blindness in children. This study describes the epidemiology and medical care associated with nonfatal pediatric (≤17 years of age) eye injury-related hospitalization in the largest industrial base for plastic toy production in China.

**Methods:**

A population-based retrospective study of patients hospitalized for ocular and orbital trauma in the ophthalmology departments of 3 major tertiary hospitals from 1st January 2001 to 31st December 2010 was performed.

**Results:**

The study included 1035 injured eyes from 1018 patients over a 10-year period: 560 (54.1%) eyes exhibited open globe injuries, 402 (38.8%) eyes suffered closed globe injuries, 10 (1.0%) eyes suffered chemical injuries and 8 (0.8%) eyes exhibited thermal injuries, representing an average annual hospitalization rate of 0.37 per 10,000 (95% confidence interval [CI], 0.36–0.38) due to pediatric eye injury in the Chaoshan region. The mean patient age was 9.2±4.4 years with a male-to-female ratio of 3.3∶1 (*P* = 0.007). Children aged 6 to 11 years accounted for the highest percentage (40.8%, 416/1018) of hospitalization, 56.7% (236/416) of whom were hospitalized for open globe wounds. Injury occurred most frequently at home (73.1%). Open globe wounds cost the single most expensive financial burden (60.8%) of total charges with $998±702 mean charges per hospitalization.

**Conclusions:**

Open globe wounds occurred at home are earmarked for the priorities to prevention strategies. Higher public awareness of protecting primary schoolchildren from home-related eye injuries should be strengthened urgently by legislation or regulation since the traditional industrial mode seems to remain the pattern for the foreseeable future. Further research that provide detailed information on the specific inciting agents of pediatric eye injuries are recommended for facilitating the development and targeting of appropriate injury prevention initiatives.

## Introduction

Ocular trauma is the leading cause of monocular visual disability and noncongenital unilateral blindness in children [1]. Increasingly, attention has been focused on the worldwide epidemic of eye injuries in the pediatric population, which carries an incidence rate of 0.746 to 9.9 per 10,000 in the United States [2], [7], [9], [13], [17], [48] and other developed countries [18], [36]. Prior epidemiologic studies of eye injury have produced varied results, in part because of differences in study design, time, and region. Population-based studies of pediatric ocular trauma have indicated that approximately two-thirds of injured patients are males, predominantly with closed globe injuries at home [7], [9]–[10], [17]–[18], [37]–[38]. However, when study subjects are restricted to eye injuries treated at any one of the healthcare centers (including tertiary hospitals), visual impairment is mainly due to open globe injuries [3], [6], [12], [29], [49]. Pediatric ocular trauma is a particular concern because injured pediatric eyes are prone to amblyopia [4], [5], [41]–[42]. From a public health and injury prevention perspective, identification of the frequency and spectrum of these injuries in a defined population and targeted educational and legislative efforts might be the tools to minimize eye injuries.

A thorough review of patients hospitalized for ocular and orbital trauma with all age groups in the Chanshan region found that 23.6% of those with eye injuries were in the 0–14 age group over a recent 10-year period (this age group makes up 21.1% of the entire population, 95%CI, 18.9–23.4) [45], resulting in a larger pediatric portion compared to other studies [6], [17], [22]. There is a paucity of population-based data available to accurately assess the magnitude of ocular trauma in children in terms of the characteristics at risk, causes of injury, environmental determinants and clinical profile despite of the limited resources in the above mention research. Data on the economic burden associated with the hospitalization of pediatric eye injuries in China is also too scarce to be compared with that from other countries such as the United States [13]. Herein, we analyzed the epidemiological characteristics of population-based nonfatal eye injuries among children 17 years of age and younger using the medical records at 3 tertiary hospitals and follow-up interviews in the largest industrial base for plastic toy production in China spanning 10 years.

The objectives of this study were as follows:

1. To identify pediatric eye injuries associated with the local toy manufacturing industry so as to offer insight into injury prevention strategies.
2. To investigate the medical care and hospital charges associated with major categories of pediatric eye injuries, which may assess the injury-related financial burden.

## Methods

### Data Source

The Chaoshan region of investigation is located in the eastern part of Guangdong Province, including 3 cities and covered approximately 10,346 km^2^. The research described herein adhered to the tenets of the Declaration of Helsinki. The ethics committees of the Medical College, Shantou University, the Joint Shantou International Eye Center, the First Affiliated Hospital of Shantou University, and the Shantou Central Hospital approved this study. The ophthalmology departments of these 3 tertiary hospitals offer eye care services for the emergency and specialized care of hospitalized patients of all ages with ocular or orbital diseases, and all hospitals house a 24-hour ophthalmic emergency department with an equivalent level of healthcare. This setup provided the opportunity to analyze ocular trauma in a well-defined study region. A population-based survey of patients admitted by the ophthalmology departments of 3 major tertiary hospitals was conducted for the period 1st January 2001 to 31st December 2010, with goals that identified all of the medical records of patients hospitalized for ocular and orbital trauma by computerized databases. Ocular trauma was defined as any injury that affected the eye or adnexa, required hospital admission, and had a principal or secondary discharge diagnosis from the International Classification of Diseases, Tenth Revision, Clinical Modification (ICD-10-CM). Attempts were made to contact all cases of ocular trauma. An interview-based informed written consent was obtained from all patients, caregivers, or guardians. All case records were kept anonymous and only patient information was extracted for research purpose. A total of 3559 participants (response rate: 98.1%, 3559/3627) with completed data was included in this study. Excluded from the study were 26 cases for refusing to consent with incomplete records and 42 lost-to-touch cases.

Eye injuries from 3559 cases were classified using the standardized international classification of ocular trauma (Birmingham Eye Trauma Terminology) [14], [15]. The extracted patient data included demographic characteristics, cause and place of injury, presentation interval after injury, clinical diagnosis, primary and secondary treatments, hospital charges and duration of hospitalization. Initial visual acuity was the best corrected Snellen visual acuity in the affected eye at the time of presentation. A complete ophthalmological evaluation was performed at 12 month post-injury with best-corrected visual acuity, slit lamp examination, fundoscopy test, final clinical diagnosis and hospital charges in the outpatient department of one of the 3 tertiary hospitals (mean duration of follow up 12.6±1.5 month). We also followed the Ocular Trauma Score (OTS) [16] in the evaluation of the final visual outcome. The above mentioned information was added to the same patients materials which from the medical records. Of all the 3559 participants at baseline, 1018 (28.6%) pediatric records were abstracted for the purposes of this study. The regional pediatric population (aged 17 years or less) was 2,727,842 (95% CI, 2,651,084–2,804,600) according to the National Statistical Yearbook 2010 during the study period, and this number was used as the denominator to calculate rates of eye injuries. A total of 295 eyes without initial visual acuity were excluded from the OTS study group because those patients were too young or presented with too serious symptoms to receive the visual acuity examination.

For meaningful comparison with previous studies, injury causes were classified in to 6 broad categories as follows: work-related injuries, home-related injuries (e.g., falls or penetrating objects, toy bullets, household objects struck against the eye), school-related injuries (e.g., stationary learning, such as injuries due to pencils or knives), sports-related injuries, road traffic-related injuries, violence-related injuries, and other various outdoor activity-related injuries that could not be classified into the former categories (e.g., injuries due to firecrackers and animal assaults ).

### Statistical Analysis

The data were analyzed using SPSS version 17.0 (SPSS, Inc., Chicago, IL, USA). Statistical analyses of the quantitative data, including descriptive statistics, and parametric and nonparametric comparisons, were performed for all variables. Frequency analyses were performed using the Χ^2^ test. A one-way analysis of variance (ANOVA) evaluated differences in parametric variables. Categorical evaluations were performed for the numeric scores, which represented the likelihood of final visual acuity in the OTS study. Correlation analyses between initial and final visual acuity and OTS and final visual acuity were performed using the Spearman rank correlation. All *P* values are 2-sided, and a *P* value less than 0.05 was considered statistically significant.

## Results

### Characteristics of Patients Hospitalized for Eye Injuries

This study included a total of 1035 injured eyes from 1018 patients over a 10-year period. The characteristics of patients who were hospitalized for the treatment of eye injuries are presented in Table 1. The average annual hospitalization rates due to eye injuries were 0.37 per 10,000 (95% CI, 0.36–0.38) in children aged 17 years or less in our study region, 0.286 per 10,000 (95% CI, 0.278–0.294) in males, and 0.084 per 10,000 (95% CI, 0.082–0.086) in females. Patient ages ranged from 2 months to 17 years, with a mean and standard deviation of 9.2±4.4 years. A total of 780 (76.7%) patients were males, and 238 (23.3%) patients were females (*P* = 0.006), which yielded a male-to-female ratio of 3.3∶1. The mean ages were 9.7±4.4 years for males and 7.6±4.2 years for females (*P* = 0.011). Children aged 6 to 11 years exhibited the highest percentage (40.8%) of hospitalizations. No significant differences in the frequency of injuries between left and right eyes were observed (520 right vs. 481 left, 17 bilateral eye injuries). Tertiary hospitals were the most common primary care centers for all eye injury-related hospitalizations (44.2), followed by secondary hospitals (28.8%). A total of 128 patients (12.6%) sought medical care more than 24 hours after an injury.

**Table 1:** Characteristics of pediatric hospitalization for eye injuries: 2001–2010.

Selected Characteristics	Male (%)	Female (%)	Total (%)
Total patients	780 (76.7)	238 (23.3)	1018 (100)
Total eye injuries	794 (76.7)	241 (23.3)	1035 (100)
Mean duration of follow up (months)			12.6±1.5
Age group, (years)			
0–5 (preschool)	167 (21.4)	91 (38.3)	258 (25.3)
6–11 (primary school)	319 (40.9 )	97 (40.7 )	416 (40.8)
12–17 (secondary school)	294 (37.7)	50(21.0)	344 (33.9)
Mean ± SD a	9.7±4.4	7.6±4.2	9.2±4.4
Annual rate of hospitalization (≤17 years) b per 10,00095% CI c	0.286(0.278–0.294)	0.084(0.085–0.09)	0.37(0.36–0.3.8)
Laterality of eyes			
Right eye	394 (50.5)	126 (52.9)	520 (51.1)
Left eye	372 (47.7)	109 (45.8)	481 (47.2)
Both eyes	14 (1.8)	3 (1.3)	17 (1.7)
Primary care within 24 hours after injury			
Non-management	81 (10.4)	22 (9.2)	103 (10.1)
Self-resolving	14 (1.8)	6 (2.6)	20 (2.0)
Community primary care	110 (14.1)	42 (17.6)	152 (14.9)
Secondary hospitals	229 (29.3)	64 (26.9)	293 (28.8)
Tertiary hospitals	346 (44.4)	104 (43.7)	450 (44.2)
Presentation interval after injury (hours)			
<6	473 (60.6)	141 (59.2)	614 (60.3)
6–12	144 (18.5)	41 (17.3)	185 (18.2)
12–24	74 (9.4)	22 (9.2)	96 (8.9)
>24	89 (11.4)	34 (14.3)	123 (12.1)

a Standard Deviation.

b Rates of injury calculated using national population estimates from the National Statistical Yearbook 2010 (China).

c 95% confidence interval.

### Clinical Diagnosis by Age and Gender

Table 2 presents the hospitalization information for the major diagnoses of eye injuries by age and gender. The majority of patients were hospitalized for open globe wounds (54.1%), followed by closed globe wounds (38.8%) and lacrimal apparatus/eyelid lacerations (7.0%). The distributions of major clinical diagnoses varied by age. Among the children patients aged 6 to 11 years, 42.2% were hospitalized for open globe wounds. Children aged 12 to 17 years exhibited a higher percentage of hospitalization for closed globe wounds (45.5%). Males experienced a greater number of hospitalizations for each type of eye injury.

**Table 2:** Diagnoses of major pediatric eye injuries by age and gender.

Type of injury	Total patients	Age groups (years )			Gender	
	Number (%)^a^	0–5 (%)	6–11 (%)	12–17 (%)	Male (%)	Female(%)
Open globe wound	560(54.1)	172(30.7)	236(42.2)	152(27.2)	401(71.5)	159(28.5)
Closed globe wound	402(38.8)	63(15.7)	156(38.8)	183(45.5)	338(84.1)	64(15.9)
Chemical burn to eyeball and adnexa	10(1.0)	6(60.0)	2(20.0)	2(20.0)	9(90.0)	1(10.0)
Thermal burn to eyeball and adnexa	8(0.8)	0	4(50.0)	4(50.0)	5(62.5)	3(37.5)
Orbital wall fractures	4(0.4)	0	2(50.0)	2(50.0)	4(100)	0
Lacrimal apparatus and eyelid laceration	72(7.0)	18(25.0)	36(50.0)	18(25.0)	52(72.2)	20(27.8)
Others^b^	18(1.7)	6(33.3)	6(33.4)	6(33.3)	16(88.9)	2(11.1)

a Percentages are over total 100.0% because one participant might have more than one type of injury according to the primary and/or secondary diagnosis.

b Includes a foreign body on the external eye, injury to the optic nerve and pathways, injury to the oculomotor, trochlear, or abducens nerve, and conjunctival injuries.

### Injury Causes by Age and Gender

Among the hospitalizations for the recorded injury causes in both genders (963 patients, 94.6%), the most frequent injury causes were home-related injuries (56.6%), violence-related injuries (14.1%), outdoor activity-related injuries (11.3%) and work-related injuries (6.9%). The majority of injuries (39.6%) occurring in males were distributed in the 6–11 age group, for which the most frequent injury types were home-related injuries (58.9%). The majority of injuries (35.7%) occurring in females were distributed in the 0–5 age group, for which the most frequent injury types were also home-related injuries (68.2%). Children with younger age groups (≤11 years old) were prone to get injured (*P* = 0.005; Pearson’s Chi-squared test).

### Injury Locales and Major Clinical Diagnoses

**Figure 1:**
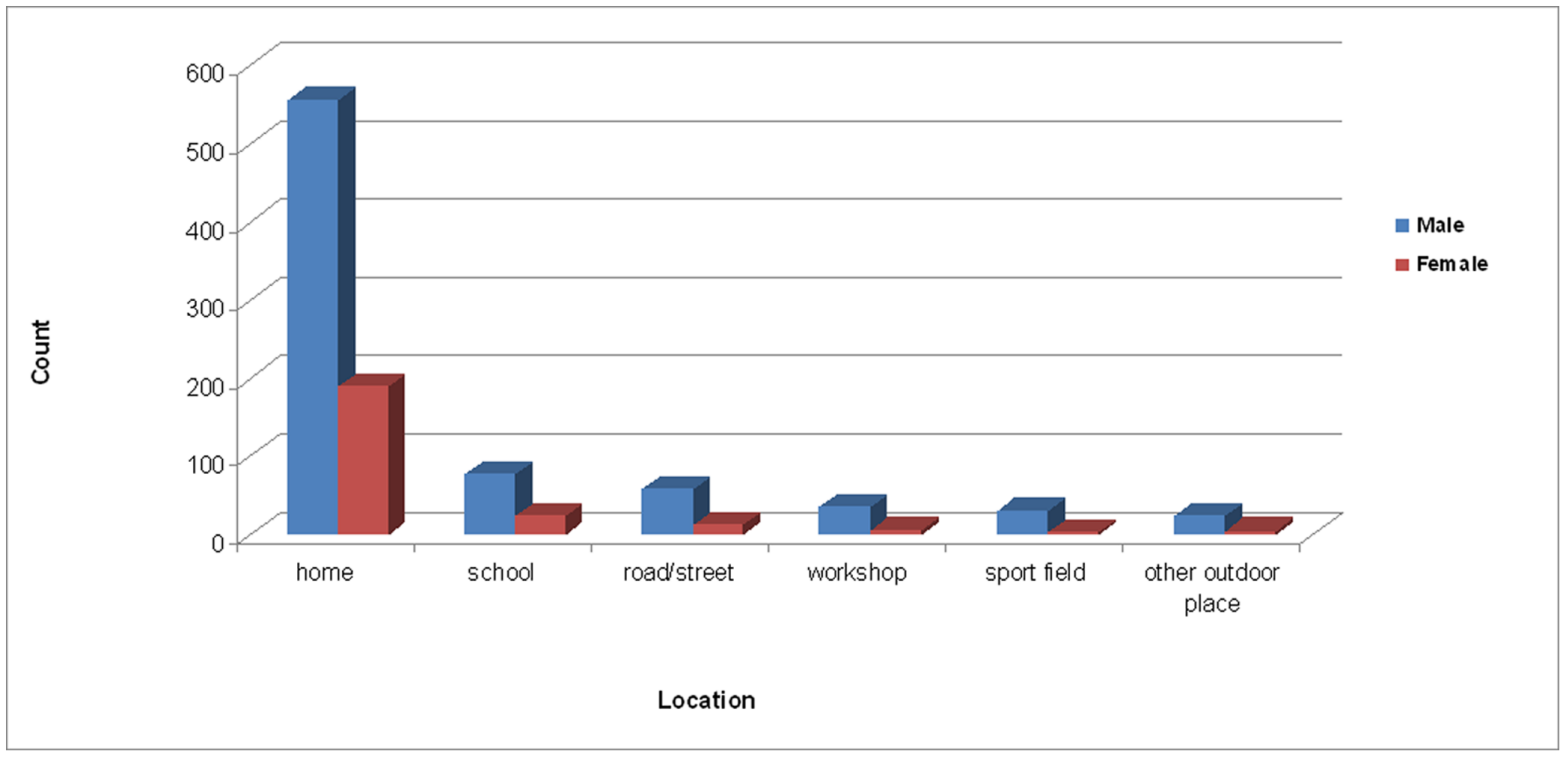
Frequencies of injury locales by gender. The most common locales of pediatric eye injury were home (73.1%), school (9.8%), and road/street (7.0%). Male children (62%) were more prone to ocular trauma in all locales than female (38%) (P<0.05).

**Figure 2:**
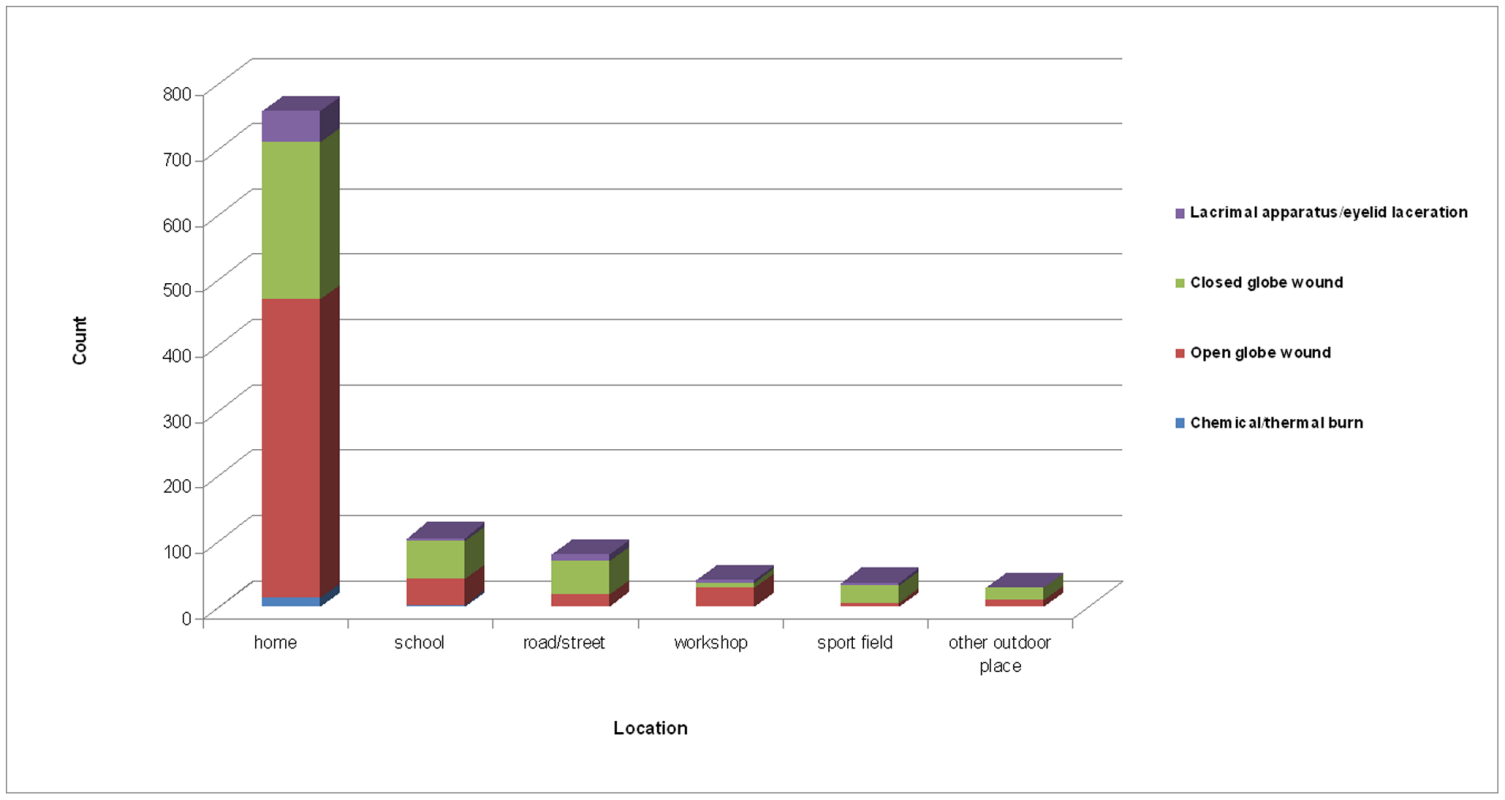
Frequencies of major clinical diagnoses of eye injuries by locale. Significant differences in the frequencies of major clinical diagnoses of eye injuries in home-related injuries were observed (61.4% open vs. 32.4% closed, 6.3% lacrimal apparatus/eyelid laceration vs. 1.9% chemical/thermal burn, P<0.001). Closed globe wounds were more frequent than open globe wounds in school- and road/street-related eye injuries (P<0.05).

Most eye injuries in our study occurred at home (73.1%). School (9.8%) and road/street (7.0%) were the next most common settings. Male children were more prone to ocular trauma than females in all settings (62% vs. 38%, *P*<0.05) (Figure 1). Significant differences were observed in the frequency of major clinical diagnoses of eye injuries at home (61.4% open vs. 32.4% closed, 6.3% lacrimal apparatus/eyelid laceration vs. 1.9% chemical/thermal burn, *P*<0.001). The frequency of closed globe wounds was greater than open globe wounds in school and road/street-related eye injuries (*P*<0.05) (Figure 2). Eye protection (conventional glasses) was present in only 5 cases (0.5%).

### Management and Visual Outcome

A total of 307 (29.7%) eyes were managed conservatively on medications, and the remaining 728 (70.3%) eyes required additional procedures (Table 3). Ocular wall repair (394, 38.1%) and lens extraction (134, 12.9%) were the most commonly required additional procedures. Enucleation was performed for globe rupturing and uncontrolled endophthalmitis in 6 (0.6%) no light perception (NLP) eyes in males. A comparison of final visual acuity and presenting visual acuity is presented in Table 4. Initial visual acuity correlated with final visual acuity (Spearman's correlation coefficient ρ = 0.659; *P*<0.001). The OTS diagnosis also correlated with final visual acuity (Spearman's correlation coefficient ρ = 0.597; *P*<0.001) (Table 5).

**Table 3:** Nonsurgical and surgical management reports from presentation to final follow up in eye injury cases by gender.

Management	Male		Female		Total	
	Number	(%)	Number	(%)	Number	(%)
Non-surgical	264	33.2	43	17.8	307	29.7
Surgical	530	66.8	198	82.2	728	70.3
Ocular wall repair	292	36.8	102	42.3	394	38.1
Lens extraction	92	11.6	42	17.4	134	12.9
Vitrectomy	36	4.5	20	8.3	56	5.4
Anterior chamber washout	38	4.8	12	5.0	50	4.8
Scleral Buckle	10	1.3	4	1.7	14	1.4
Keratoplasty	0	0	0	0	0	0
Enucleation	6	0.8	0	0	6	0.6
Glaucoma surgery	8	1.0	2	0.8	10	0.9
Canalicular anastomosis	46	5.8	16	6.6	62	6.0
Obital fracture repair	2	0.2	0	0	2	0.2
Total	794	100	241	100	1035	100

**Table 4:** Final visual acuity compared with presenting visual acuity.

Visual acuity	At presentation		Final visual acuity^c^	
	Frequency	(%)	Frequency	(%)
NLP	39	3.8	37	3.6
LP-1/50	286	27.6	103	9.9
1/50–1/20	148	14.3	91	8.8
1/20–0.3	114	11.0	146	14.1
≥0.3	153	14.8	398	38.5
Others	295^a^	28.5	260^b^	25.1
Total	1035	100	1035	100

a Includes the patients too young to receive the visual acuity examination and those with presenting symptoms too serious to apply to the visual acuity examination.

b Includes the patients too young to receive the visual acuity examination, those transferred to another health care facility or receiving home health care, and those with missing/unrecorded final visual acuity due to loss of follow-up (55 children).

c Final visual acuity (FVA) is the presented visual acuity of patients at last follow up in the outpatient department of one of the 3 tertiary hospitals, (Spearman's correlation coefficient ρ = 0.659, *P*<0.001 ).

**Table 5:** Correlation of the final visual acuity category with the OTS in the OTS study group (740 eyes).

OTS		Final visual acuity category					
Raw score	Category	NLP(%)	LP-1/50 (%)	1/50-1/20(%)	1/20-0.3(%)	≥0.3 (%)	Total (%)
0–44	1	8(1.1)	35 (4.7)	5 (0.7)	22 (3.0)	26(3.5)	96 (3.0)
45–65	2	11 (1.5 )	23 (3.1 )	24(3.3)	32(4.3)	103(13.9)	193 (26.1)
66–80	3	8(1.1)	26 (3.5 )	38 (5.1)	52(7.0)	182 (24.6)	306 (41.3)
81–91	4	7(0.9)	7(1.0)	17(2.3)	19(2.6)	37 (5.0)	87 (11.8)
92–100	5	3(0.4)	5(0.7)	4(0.5)	13(1.7)	33(4.5)	58 (7.8)
Total		37(5.0 )	96 (13.0 )	88 (11.9 )	138 (18.6 )	381(51.5)	740 (100)

(Spearman's correlation coefficient ρ = 0.597, *P*<0.001).

### Medical Care Charges

The estimated total charges for pediatric eye injury-related hospitalization in the study region from 2001 to 2010 were $918,986 (Table 6). Open globe wounds accounted for the largest percentage of total hospital charges (60.8%). The largest mean charges per hospitalization were caused by orbital wall fractures ($1018±516). Length of stay for the majority of patients in the hospital for all pediatric eye injury-related hospitalization was more than 15 days (869, 85.4%), with a mean stay of 8.2±6.5 days. The majority of hospitalized patients with eye injuries were routinely discharged (932, 91.6%).

**Table 6:** Medical care characteristics of hospitalization for pediatric eye injuries by diagnosis.

Medical care characteristics	All injuries	Open globewound	Closedglobe wound	Chemical burn to eye and adnexa	Thermal burn to eye and adnexa	Orbital wallfracture	Lacrimal apparatus andeyelid laceration	Others^a^
Total hospital charges : $	918986	558835	312695	5361	4692	4073	31823	1607
% of total hospital charges	100	60.8	34.0	0.6	0.5	0.4	3.5	0.2
Mean charges per hospitalization: $	903	998	774	536	939	1018	439	90
SD^b^	662	702	598	144	116	516	66	51
Length of stay, (days)								
0–7	49	280	290	5	5	1	51	13
8–14	100	164	81	3	1	2	16	3
≥15	869	116	31	2	2	1	5	2
Mean stay, (days)	8.2	8.9	7.3	7.6	9.7	10.5	6.8	5.6
SD^b^	6.5	5.7	7.0	3.1	6.2	5.5	0.5	3.7
Discharge status								
Routine	963	549	369	9	5	3	26	2
Others^c^	55	11	33	1	3	1	4	2

a Includes a foreign body on the external eye, injury to the optic nerve and pathways, injury to the oculomotor, trochlear, or abducens nerve, and conjunctival injuries.

b Standard Deviation.

c Includes transfer to a short-term hospital or another health care facility, home health care, against medical advice, missing, and not recorded.

## Discussion

### Principal Findings of the Study

Because a nationwide eye injury surveillance system has not been established in China, hospital-based medical records can constitute a valuable source of epidemiologic information. This large-sample retrospective study provides an in-depth analysis of nonfatal pediatric eye injury-related hospitalizations in the southeast coastal industrial region of China. Our results underscore that ocular trauma causes significant visual impairment and financial burden in the pediatric population particularly aged younger than 11 years. Open globe wounds occurred at home are earmarked for the priorities to prevention strategies. Higher public awareness of supervision of primary schoolchildren should be strengthened as an urgent need by legislation or regulation. Increasing awareness of the serious nature of home-based manufacturing environment would help to develop a comprehensive plan for educating both parents and schoolchildren to minimize preventable pediatric eye injuries. The current findings might be considered as a baseline for future research on regional eye injuries.

### Comparison with other Studies

A comparison of the major characteristics of pediatric eye injuries from different countries is presented in Table 7. This study estimates that the annual incidence of hospitalization for pediatric eye injuries is 0.37 per 10,000, which is less than that reported by Megan Brophy et al. in the United States in 2006 (0.89 per 10,000) [13], Caroline J MacEwen et al. in Scotland in 1999 (0.885 per 10,000) [18] and Ksenija Karaman et al. in Croatia in 2009 (2.25 per 10,000) [36]. Underestimation of true ocular trauma incidence may have occurred due to loss of patients with minor trauma, who could have sought care for their eye injuries in hospitals other than the ones under study, and loss of polytraumatized cases. The long-term nationwide eye injury registration system is recommended for establishment of prevention measures.

**Table 7:** A comparison of reported characteristics of pediatric eye injuries by different countries.

Year of publication	Country ^[ref]^	Study location	Study design	Sample N^a^(95%CI) e	Definition of children age (yrs)	Incidence rate(per 10,000)	Variable with the leading percentage				
							M/F	Clinical diagnosis (%)	Locales (%)	Age group (yrs) (%)	Injury causes (%)
2012	USA [^7^]	National survey	Retrospective- cohort	1948500	<18	1.431	1.6∶1	Contusion/abrasion(53.7)	Home (65.8)	15∼17 (NA)	Being struck by or against an object (56.6)
	USA [^9^]	National survey (CPSC-NEISS)^c^	Retrospective	1406200	<18	9.9	1.9∶1	Contusion/abrasion(45.0)	Home (69.4)	≤4 (32.0)	Contact with the product in activity- and consumer product-related (75.0)
	Nepal [^10^]	Population-based	Retrospective	554	<16	NA f	1.6∶1	Sub-conjunctival hemorrhage(17.3)	Home (36.8)	5–10 (38.1)	NA
	Our research	Population-based	Retrospective	1018	<18	0.37 b	3.3∶1	Open globe injuries(54.1)	Home (73.1)	6–11 (40.8)	Exposure in the household manufacturing
2011	USA [^17^]	National survey(CPSC-NEISS)^c^	Retrospective	5929(4956–6897)	≤18	0.746	1.7∶1	Dermatitis/conjunctivitis (36.0)	Home (71.0)	0∼4 (47.3)	Spray paint (10.0)
	Egypt [^3^]	One hospital unit	Interventional-	150	≤16	NA	2.4∶1	Open globe injuries(67.3)	Street (54.7)	2–7(50.7)	Playing(NA)
			non-comparative								
	Qatar [^11^]	One tertiary care	Retrospective- cohort	106	<16	NA	3.4∶1	Closed globe injuries(59.4)	Home (42.5)	≥5(58.5)	NA
2010	Turkey [^6^]	One hospital unit	Retrospectively	114	<16	NA	3.3∶1	Open globe injuries(72.0)	Street (57.0)	NA	Metallic substance in unsupervised play(35.1)
	Taiwan [^12^]	Population-based	Retrospective	156	<16	NA	2.1∶1	Open globe Injuries(71.2)	Home (15.4)	0∼5 (NA)	Scissors (13.5)
2009	Canada d [^28^]	One tertiary care	Retrospective- cohort	149	≤18	NA	2.7∶1	NA	Home (49.0)	5–9(34,2)	Playing (45.0)
	Croatia[^36^]	National survey	Retrospective	106	<18	2.25 b	5.2∶1	Closed globe injury(89.89)	Outdoors (58.5)	14∼18(NA)	Blunt objects (30.3), Missiles (24.2)
	Nigeria[^25^]	One hospital unit	Retrospective	62	<16	NA	2.3∶1	Closed globe injuries(87.1)	NA	6∼10(41.9)	Twigs and farming activities(−)
	Kuwait [^31^]	One tertiary center	Case series non-comparative	19	<18	NA	8.5∶1	Hyphema (100)	NA	6∼10(NA)	Toy-gun pellets(NA)
2008	USA [^24^]	National survey (CPSC-NEISS)^c^	Retrospective	512079	≤12	NA	1.7∶1	Abrasion/contusion(44.6)	Outdoors (NA)	9∼12 (29.0)	Sport-related (13.5)
	USA [^38^]	National survey(CPSC-NEISS)^c^	Retrospective	85800	≤19	NA	3.3∶1	Burns (60.2)	Home (NA)	10∼14 (NA)	Firecracker in fireworks-related eye injuries (29.6)
	Taiwan [^46^]	One hospital unit	Retrospective	228	<16	NA	2.0∶1	Closed globe injury (78.1)	NA	4∼6 (35.5)	Falling(−)
2007	Brazil[^8^]	One hospital unit	Retrospective	273	<16	NA	NA	Closed globe injuries(73.6)	Home (53.1)	7∼10(39.9)	External agents like stone, iron etc. (27.9)
2006	USA [^13^]	National survey	Cross-sectional	3834	≤20	0.89 b	2.3∶1	Open wound of theocular adnexa(25.9)	NA	18–20 (23.7)	Motor vehicle crash (28.8)
2003	Colombian[^37^]	State survey	Retrospective	393	<16	NA	1.8∶1	Closed globe injuries(82.4)	Home (44.4)	6–10(63.3)	Blunt objects (35.1)
	USA[^41^]	Observationalcase series	Retrospective- case series	16	≤18	NA	NA	Periocular abrasions, edema (100)	NA	3∼16(NA)	Airbag-associated ocular trauma
2002	Romanian[^49^]	Hospital unit(s)	Retrospective	138	NA	NA	3.6∶1	Open globe injuries	Home (25.0)	9∼10(NA)	Playing toys (46.0)
	India [^29^]	One tertiary care	Prospective	204	≤14	NA	1.9∶1	Open globe Injuries (53.9)	Outdoors (49.5)	≥5(87.7)	unsupervised games like bow,arrow (15.2)
2000	USA [^40^]	National survey	Retrospective- case report	103730	NA	NA	NA	Fracture of orbital floor, nasal structures, zygoma(−)	Sports field (NA)	NA	Baseball accounted for 40% of eye injuries
1999	Scotland [^18^]	Population-based	Prospective observational	93^−^	≤14	0.885 b	2.7∶1	Blunt trauma (65.0)	Home (51.0)	5∼14(NA)	Sports (16.0)
1998	Spain[^50^]	One hospital unit	Retrospective	257	NA	NA	4.0∶1	Closed globe injuries (85.6)	School-home(33.0)	NA	Playtime-leisure(32.0)
1997	USA [^44^]	One Children’s Hospital	Retrospective- descriptive	166	≤18	NA	2.4∶1	NA	Home (77.0)	10∼14(54.2)	Intentional injuries by nonpower firearm injuries (49.0)
1997	Hongkong[^27^]	One hospital unit	Retrospective	60	<12	NA	2.8∶1	Contusion (48.3)	Outdoors(NA)	3∼5 (78.0)	Common household items(22.0)
1990	USA [^48^]	State survey	Retrospective	NA	≤14	1.52	4.0∶1	Closed globe injuries(NA)	NA	11∼15(NA)	Accidental blows and falls (37.0).

a total number of patients, not injured eyes.

b hospitalization rate.

c CPSC-NEISS, Consumer Product Safety Commission’s National Electronic Injury Surveillance System.

d Patients presenting were identified using the Canadian Hospital Injury Reporting and Prevention Program(CHIRPP).

e 95% confidence interval.

f not available (NA).

The distribution by age group revealed that 40.8% of children aged 6 to 11 years were alone or without adult supervision at the time of eye injury. The results of this current study are similar to previously studies in other countries, which showed that male children below the age of 10 years were predominant [8], [10], [25], [28], [31], [37], [49]. This could be interpreted on the basis that primary schoolchildren are more susceptible than the preschool-aged group, although they are still relatively immature, these children are slightly more independent which may make them more vulnerable [18]–[22]. Worthy of note is that the commonest locale for injury occurrence was the home, accounting for 73.1% of all accidents in the current research. The proportion is higher than which reported in the aforementioned literatures concerning all types of eye injury, ranging from 15.4% to 69.4% [7]–[12], [18], [28], [37], [49]. Many of these risk factors remain unrecognized by the retrospective study design because evaluation of the home-related causes mainly requires a thorough history, preferably from the child. The child might withhold full disclosure of detail if he or she feels responsible for the accident. Likewise, too young children could not provide enough information of exact time, place, and agents of injury which is critical to appropriate assessment and treatment. For these reasons, results and conclusions from retrospective studies must be interpreted cautiously. A potential explanation for the higher risk at home might be that due to the prosperity of plastic toy industry, there are many household toy manufacturing plants in the Chaoshan region. The toys of substandard quality or domestic utensils can be found in any home, which imposes potential danger for children. This hypothesis is supported by the previous report of patients with all age groups, which found that knives/scissors and toys bullets remained the leading (641, 89.7%) agents of the home-related eye injuries according to the recorded injury causes [45]. These factors might also contribute to the relatively larger portion of preschool children in the total pediatric patients (258/1018, 25.2%) contrary to the injury rate of majority in secondary schoolchildren reported by previous studies [7], [13], [36], [44], [48].

Furthermore, children less than 11 years experienced a higher rate of hospitalization for open globe wounds (72.9%), which differs from most previous studies in which closed globe wounds exhibited the highest proportion [7], [10], [11], [23]–[27], [36], [37]. Several explanations may account for the differences in the data reflection. First, all cases in our study were treated in hospitals as inpatients and the cases in the other reports included those treated in other health care settings [25], [50] or emergency departments [7]–[9], [24]. Children admitted to the hospitals may have more severe injuries than those treated in other settings (e.g., private physician offices, hospital clinics, or emergency departments). The composition and sample size can likely be attributed to statistical differences in part. Additionally, in the Chaoshan culture, schoolchildren are allowed and even encouraged to assist in toy manufacturing without protective eyewear, which is one of the major risk factors for work-related eye injuries. Children often have less coordination, reduced ability to operate accurately, lessened emotional control, and deficient awareness for self-protection as compared to adults. It is particularly important to counsel parents to supervise their children and withdraw them from any risk activities (e.g., playing sharp toys or using power tools) since work-related injuries are still one of the frequent injury causes in our pediatric population (6.9%). A significantly declined incidence rate of occupational injuries and sport-related injuries in the developed countries has verified the efficacy of a combination of sufficient education and legislation [2], [18], [43]. Higher public awareness of correct first-aid treatments should also be strengthened as an urgent need to educate preschool staffs recently [23]. The targeted public health campaigns could be appropriately designed according to our own experience, even though all these measures will go a long way in decreasing the incidence of trauma related ocular morbidity in children.

Open globe wounds exhibit a poorer visual prognosis than closed globe wounds, because they are more likely to require surgeries and result in long-term visual impairments or blindness and concomitant developmental delays [32], [46], [47]. Higher frequency of this kind of clinical diagnoses could likely cause a serious prognosis in our pediatric patients, presenting final visual acuity with no light perception in 3.6% and light perception to 20/200 in 24.8% of patients. Therefore, 28.4% of the children should be defined as legally blind according to the legal definition of blindness in the United States (best corrected visual acuity of 20/200, US notation; 6/60, 6 m notation; or 1 logMar in the better eye) [30]. Good initial vision and a high OTS were statistically correlated with good final vision in our study and were well proven by other reports [22], [32]–[35] although these values are not clinically used in China. Visual prognoses in children remain worse than adults despite therapeutic advances because of the nature of the injuries and amblyopic problems. A total of 94 eyes (9.1%) were associated with injuries to the lacrimal apparatus and eyelid laceration, orbital wall fractures, or other noneyeball structures in this research. Eyelid lacerations often occur in conjunction with other facial injuries [40]. Orbital fractures in children are more likely to cause entrapment of orbital contents due to the structure of orbital bones at an early age and require earlier surgical repair than adult population [39]. More serious orbital injuries resulted in severe neurologic damage from other head injuries sustained, as well as significant permanent vision loss, which demonstrates that ocular trauma requires a neurosurgical approach. Therefore, we may conclude that ocular trauma was a significant cause of visual impairment in this pediatric population. Likewise, a further prospective analysis needs to be performed in order to understand inciting agents vs. type of eye injuries vs. age group.

The financial and medical care characteristics associated with pediatric eye injury-related hospitalizations were also investigated. To our knowledge, this study was the first to estimate the total charges associated with hospitalizations for pediatric eye injuries in China industrialized cities. The mean charges per hospitalization for pediatric eye injury were more than $906 during 2001–2010, which was a large percentage (61.6%) of the reported $1471 of per capita disposable income of the local urban residents according to the National Year Book 2010. Furthermore, the length of hospital stay in China was relatively longer than the results reported by Megan Brophy et al. in the United States in 2000 (≥15 days, 85.4% vs. 0–3 days, 77.8%) [13]. Despite of the dissimilarity in medical level, the longer stay might be mainly due to a high number of ocular wall repair in our study (38.1%, 394/1018), which are more serious and costly. The mean length of stay for closed globe wounds per hospitalization was 7.3 days, which is also longer than the stays reported in Kuwait (3.6 days) [31]. These results indicate that ocular trauma causes a significant burden of ophthalmic disease in this region.

### Limitations of the Study and Future Research

The results of this study should be considered in light of several limitations. This study underestimates the actual number of hospitalized pediatric eye injuries because only injuries treated in the tertiary hospitals were included. This study also excluded patients who might have suffered minor self-resolving ocular trauma and patients who were treated in community primary care facilities, physician offices and the ophthalmology wards of secondary hospitals. The abovementioned medical facilities do not have ophthalmic microsurgical equipment and could not handle complex medical treatment or perform surgical procedures under general anesthesia for ocular trauma. Children who required primary care in these hospitals would be referred to one of the 3 tertiary hospitals in our study, such as a ratio (43.7%) of first presentation in community care and secondary hospitals in the current results. Some individuals with ocular trauma who did not seek medical treatment for unknown reasons and patients who sought medical care out of our study region were not included.

We developed broad categories of injury causes by injury locale to provide meaningful comparisons with previous research, but these categories did not provide sufficient information to determine which inciting agents are the most dangerous in terms of producing more pediatric trauma associated with permanent vision loss. Future research should focus on more specific agents of injuries, such as open globe injuries and violence-related injuries. Additionally, medical data reported in our research are limited by the detail provided in the patient’s medical record. Hospital-based surveillance also cannot provide information on the final outcome of the injured child. The injury-related financial information would be updated by in-detail data which were abstracted from other healthcare settings, including emergency departments, primary care physicians, ophthalmologist offices, and other eye care practioner offices.

However, these limitations do not significantly affect the major findings of this study. The current results assess the descriptive epidemiology of children with eye injuries presenting to the hospitalization in the southeast coastal region of China. Further research that provide detailed information on the specific inciting agents of pediatric eye injuries are needed for facilitating the development and targeting of appropriate injury prevention initiatives. We will continue to investigate the epidemiology of work-related eye injuries and road traffic-related eye injuries because these 2 categories of eye injuries are the primary public health problems in China.
